# Cell-based and cell-free immunotherapies for glioblastoma: current status and future directions

**DOI:** 10.3389/fimmu.2023.1175118

**Published:** 2023-05-25

**Authors:** Mingming Wang, Xiaojie Wang, Xiaoyan Jin, Jingjing Zhou, Yufu Zhang, Yiyuan Yang, Yusi Liu, Jing Zhang

**Affiliations:** ^1^ Department of Cell Biology and Genetics, Medical College of Yan’an University, Yan’an, Shaanxi, China; ^2^ Basic Medical School, Shenyang Medical College, Shenyang, Liaoning, China; ^3^ Department of Hepatobiliary Surgery, the Affiliated Hospital of Yan’an University, Yan’an, Shaanxi, China

**Keywords:** temozolomide, cell-based immunotherapies, cell-free immunotherapies, treatment strategy, glioblastoma

## Abstract

Glioblastoma (GBM) is among the most fatal and recurring malignant solid tumors. It arises from the GBM stem cell population. Conventional neurosurgical resection, temozolomide (TMZ)-dependent chemotherapy and radiotherapy have rendered the prognosis of patients unsatisfactory. Radiotherapy and chemotherapy can frequently induce non-specific damage to healthy brain and other tissues, which can be extremely hazardous. There is therefore a pressing need for a more effective treatment strategy for GBM to complement or replace existing treatment options. Cell-based and cell-free immunotherapies are currently being investigated to develop new treatment modalities against cancer. These treatments have the potential to be both selective and successful in minimizing off-target collateral harm in the normal brain. In this review, several aspects of cell-based and cell-free immunotherapies related to GBM will be discussed.

## 1 Introduction

As the most common type of primary intracranial tumor, gliomas develop from a variety of neuroglial cells in the brain. According to the 2016 World Health Organization (WHO) histopathological and clinical criteria, gliomas are classified as grades I-IV (1). The use of Roman numerals in the intra-tumor grading system raises the risk of confusion between ‘II’ and ‘III’ or ‘III’ and ‘IV’, and uncorrected typographical errors may compromise treatment outcomes (2). The WHO Central Nervous System (CNS) 5 of 2021 recommends grading using Arabic numerals, where WHO grade 1 gliomas are usually considered benign, curable by complete surgical excision and rarely evolve into more advanced lesions (3). In contrast, WHO grade 2 or 3 gliomas (mesenchymal astrocytomas and mesenchymal/malignant gliomas) are aggressive, progress to more advanced lesions and have a poorer prognosis (1, 2, 4). WHO grade 4 tumors are highly malignant and present with a poor prognosis (4).

Glioblastoma (GBM) has been described as a grade 4 tumor by the WHO and is among the most fatal and recurring malignant solid tumors to date (5) accounting for 57% of all gliomas and 48% of primary CNS malignancies (6). The median survival of GBM patients is 14.6 months. GBM is presumably caused by Glioblastoma stem cells (GSC), which have rapid self-renewal and a high rate of appreciation, and decreasing GSC is useful in limiting the progression of GBM (7, 8). Since 2005, the Food and Drug Administration (FDA) has authorized only two medications and one device for the treatment of GBM, namely temozolomide (TMZ) (9), bevacizumab (BVZ) (10) and Therapeutic Tumor Fields (TTFields) (11). The prognosis of GBM patients is still unsatisfactory despite decades of efforts and advances in surgery, radiotherapy and chemotherapy. The reason for this result is directly linked to the tumor immune microenvironment of GBM. Due to the existence of the blood-brain barrier, there are very few immune cells from the blood circulation in the brain parenchymal under physiological circumstances (12). When a tumor forms, multiple types of immune cells can move to the tumor region, either to exhibit antitumor effects or to be affected by tumor cells to create an immunosuppressive phenotype while cause suppressive functions (13, 14). At this period, inflammatory variables rule the suppressive immune microenvironment of GBM, which promotes tumor development. This discouraging clinical outcome has made GBM an urgent topic for cancer research. Here, we will discuss the progress made by immunotherapy in the treatment of GBM in recent years.

**Figure 1:**
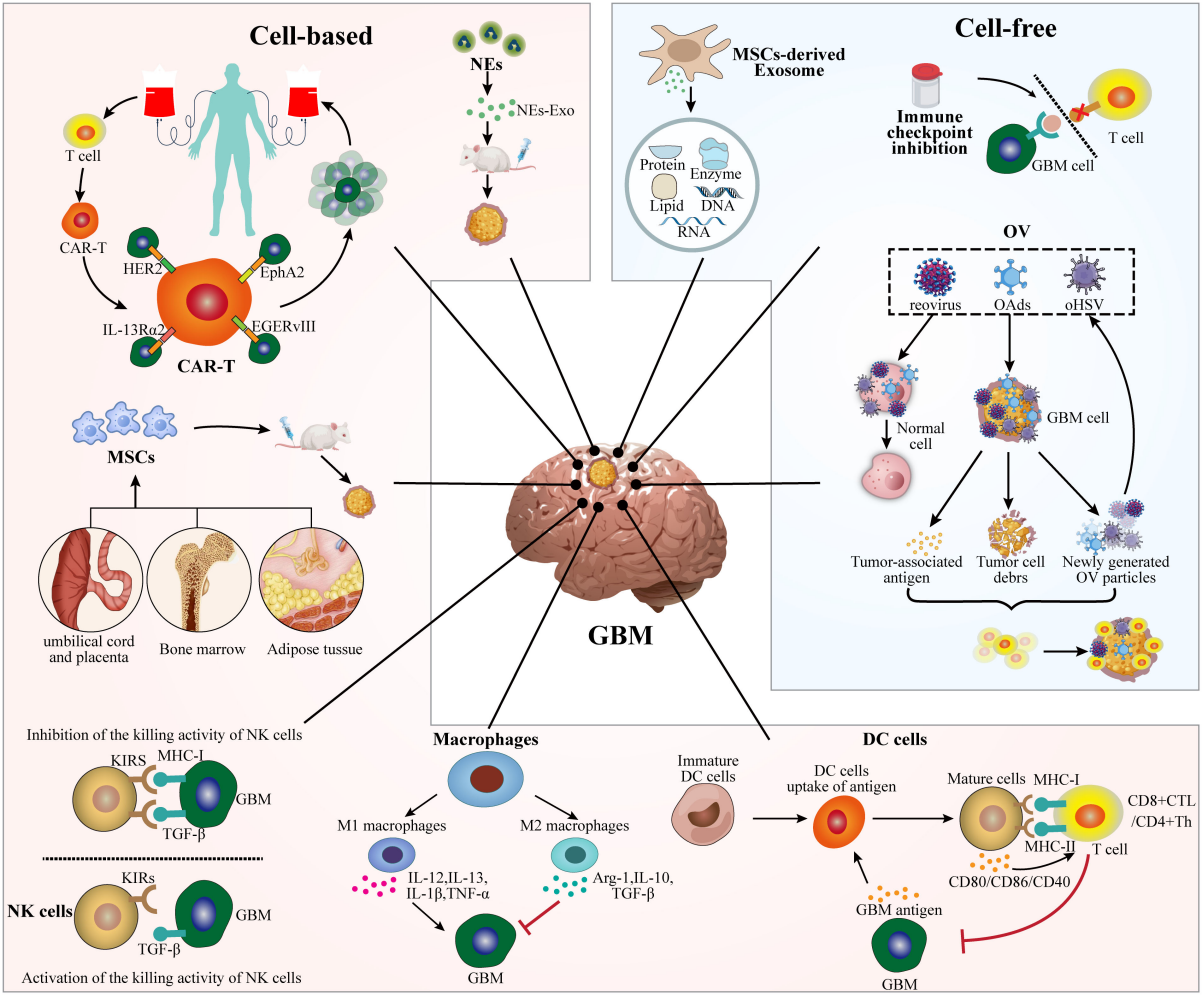
Schematic representation of cell-based and cell-free immunotherapies for GBM.

As early as the mid-nineteenth century, it was proposed that cancer treatment could be achieved by modulating the body’s immune system to combat cancer (15). Its distinct scientific and clinical benefits have given rise to the idea of immuno-oncology, which is to enhance the immune response to tumor cells through the adaptive or innate immune system, eliminating them while reducing collateral damage (16). The evaluation of the therapeutic effect of glioma has always been a difficult clinical problem. RANO/iRANO proposed by Harvard Medical School has been recognized by the neurooncology community as a therapeutic response evaluation standard for high-grade glioma, and has also become a common evaluation standard for high-grade glioma clinical trials (17). Both criteria have significant characteristics. The limitation of RANO criteria is that if patients receive immunotherapy, their immune response is different due to different constitutions. However, iRANO standard does not require a large number of case verification, but is constantly discussed and verified by experts in clinical practice. This is why immuno-oncology medications, including cellular treatments, oncolytic virus immunotherapy, and immunological checkpoint blockade therapy are being researched intensively. In this paper, a wide range of possibilities for a new generation of cell-based and cell-free immunotherapies is demonstrated, such that the recent history of GBM immunotherapy can be summarized (**Figure 1**).

## 2 Cell-based Immunotherapies for GBM

### 2.1 CAR-T cell therapy

**Figure 2:**
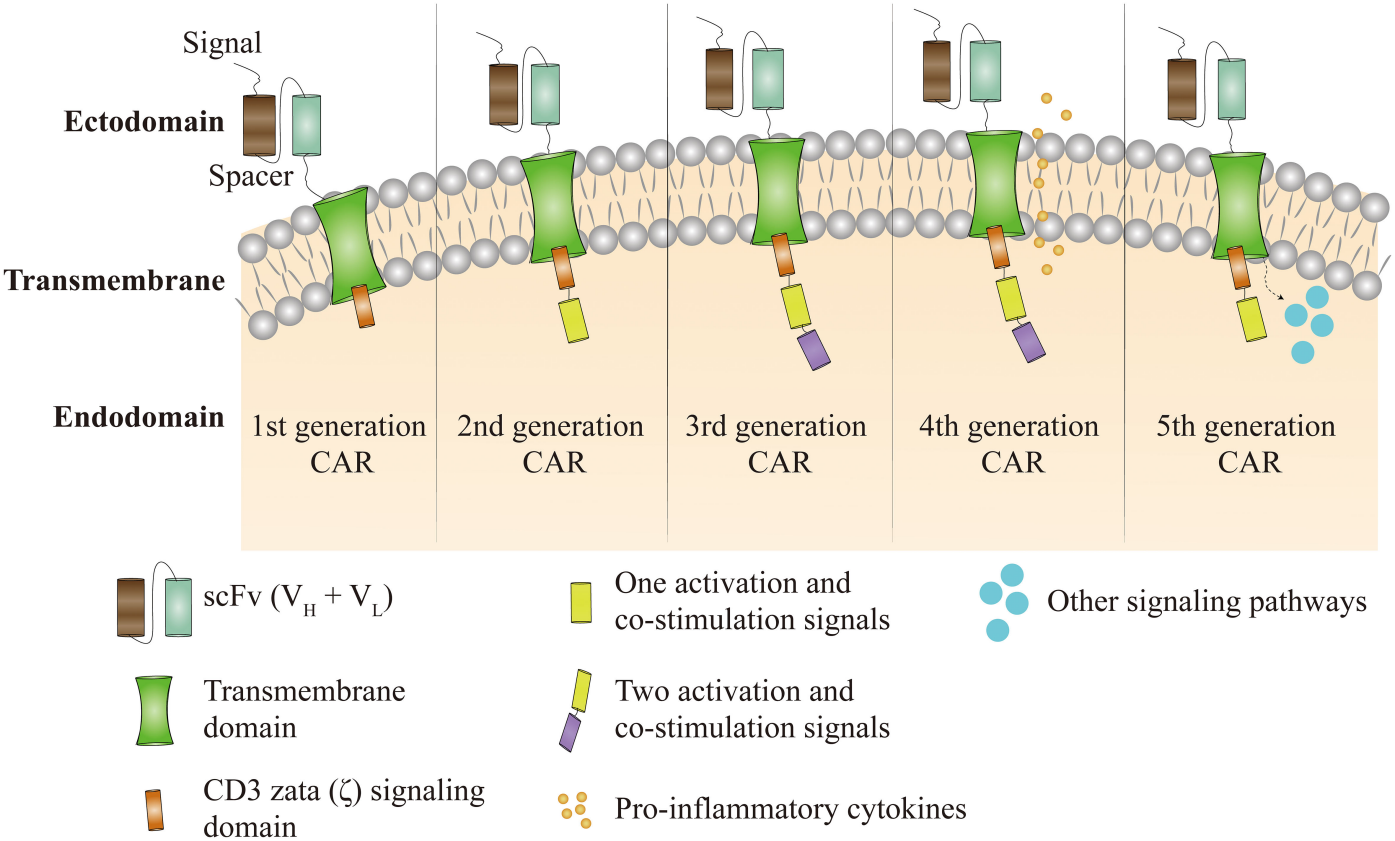
Figure 2 CAR-T cell therapy has been through five technical generations. The first generation of CAR depended only on CD3ζ to activate T cells. Clinical effectiveness is limited by a lack of intracellular co-stimulatory signaling, which prevents persistent T-cell proliferation and long-term anti-tumour effects. To the first generation CAR, the second generation CAR incorporates activation and co-stimulatory signals such as CD28 or CD137. Third generation CAR supplement first generation CAR with two co-stimulatory and activation signals, such as CD28, CD137, CD134, and OX40. Based on the third generation of CAR, the fourth generation of CARs incorporates pro-inflammatory cytokines like as IL-12 and co-stimulatory ligands with the goal of overriding tumor immune microenvironment suppression. The fifth generation of CAR is still under development and is based on the second generation of CAR with the inclusion of co-stimulatory structural domains that activate additional signaling pathways.

Chimeric antigen receptor (CAR) T cell therapy holds one of the most promise as an anti-cancer therapeutic technology. CAR are synthetic molecules formed by four regions, the antigen recognition structural domain (variable region of monoclonal antibodies with a single chain variable fragment scFv), the extracellular hinge region, the transmembrane structural domain (consisting of a hydrophobic α-helix across the cell membrane) and the intracellular T cell signaling structural domain (zeta ζ signaling chain) (18) intended to guide T cells to particular antigens. First generation CAR is a fusion protein consisting of an extracellular antigen-binding domain, generally in the form of a single-chain variable fragment of an antibody, attached to an intracellular signaling domain, most often the CD3ζ chain of the T cell receptor (TCR) (18). In second-generation CAR, the activity of CAR-T cells is enhanced through the addition of co-stimulatory structural domains fused to CD3ζ, such as CD28 or CD137 (also referred to as 4-1BB), and the involvement of these intracellular signaling domains improves anti-apoptosis, cytokine secretion, T cell proliferation and *in vivo* persistence (19). Third generation CARs that incorporate multiple co-stimulatory structural domains (e.g. CD28-41BB, CD28-OX40), have also been developed (19). Fourth generation CAR, also known as TRUCK or armored CAR, have been further augmented with factors that enhance anti-tumor activity, persistence and T cell expansion. These potentially include cytokines such as IL-2, IL-5, IL-12, enzymes that degrade the extracellular matrix of solid tumors and co-stimulatory ligands (20). The fifth generation CAR is based on the second generation CAR with the addition of co-stimulatory structures and domains that activate other signaling pathways and is still in the development stage (**Figure 2**).

CAR-T cells were first used to treat hematological malignancies and have shown remarkable efficacy (21, 22). For example, the FDA has authorized two medicines for the treatment of hematological malignancies. The first one is Tisagenlecleucel for treating B-cell acute lymphoblastic leukemia (21) and the other is Axicabtagene Ciloleucel for treating large B-cell lymphoma. These CAR-T cells target CD19 on B cells to induce effective tumor cell death (22). Given the extraordinary success in hematological malignancies, CAR-T therapy in solid tumors has also been a rapidly developing research hotspot in recent years. These include interleukin 13 receptor alpha 2 (L13Rα2), epidermal growth factor receptor variant III (EGFRvIII), type A liver ligand protein receptor 2 (EphA2) and human epidermal growth factor receptor 2 (HER2), all of which have been tested as targets for several clinical CAR-T cell therapies (**Table 1**). However, identifying good antigens in solid tumors is always a challenge, and such antigens have to be highly expressed on most cancer cells but virtually absent in normal tissue (23). Under such conditions, CAR-T cells can’t be efficiently transported to the center of solid tumor masses, and the adverse tumor microenvironment (TME) inhibits T cell activity (24).

**Table 1:** CAR-T cell based clinical studies in GBM patients that have been completed or are ongoing.

Molecular target	Clinical trial NCT number and title	Study phase	Interventions	Study Results	Sponsor/Collaborators
IL13Rα2	NCT04003649 Evaluate IL13Rα2-Targeted Chimeric Antigen Receptor (CAR) T Cells Combined with Checkpoint Inhibition for Patients with Resectable Recurrent Glioblastoma	I	Biological: IL13Ralpha2-specific Hinge-optimized 4-1BB-co-stimulatory CAR/Truncated CD19-expressing Autologous TN/MEM Cells Biological: Ipilimumab Biological: Nivolumab	Recruiting	City of Hope Medical Center (National Cancer Institute)
	NCT04661384 Evaluate IL13Rα2-Targeted Chimeric Antigen Receptor (CAR) T Cells for Adult Patients with Leptomeningeal Glioblastoma, Ependymoma or Medulloblastoma	I	Biological: IL13Ralpha2-specific Hinge-optimized 41BB-co-stimulatory CAR Truncated CD19-expressing Autologous T-Lymphocytes	Recruiting	City of Hope Medical Center (National Cancer Institute)
	NCT02208362 Cellular ImmunoTx Using Memory Enriched T Cells Lentivirally Transduced to Express an IL13Rα2-Specific, Hinge-Optimized, 41BB-Costimulatory Chimeric Receptor and a Truncated CD19 for Pts with Rec/Ref MaligGlioma	I	Biological: IL13Ralpha2-specific Hinge-optimized 4-1BB-co-stimulatory CAR/Truncated CD19-expressing Autologous TN/MEM Cells Biological: IL13Ralpha2-specific Hinge-optimized 41BB-co-stimulatory CAR Truncated CD19-expressing Autologous T-Lymphocytes	After treatment with IL13Rα2-targeted CAR T cells, GBM regression was observed, and this clinical response persisted for 7.5 months (doi: 10.1056/NEJMoa1610497)	City of Hope Medical Center (National Cancer Institute; Food and Drug Administration)
EGFRvIII	NCT02209376 Autologous T Cells Redirected to EGFRVIII-With a Chimeric Antigen Receptor in Patients With EGFRVIII+ Glioblastoma	I	Biological: CART-EGFRvIII T cells	After 18 months of follow-up, only one of ten treated GBM patients exhibited residual stable disease (doi: 10.1126/scitranslmed.aaa0984)	University of Pennsylvania (University of California, San Francisco)
	NCT03726515 EGFRvIII-Directed CAR T Cells Combined With PD-1 Inhibition in Patients with Newly Diagnosed, MGMT-Unmethylated Glioblastoma	I	Biological: CART-EGFRvIII T cells Biological: Pembrolizumab	Completed	University of Pennsylvania
	NCT01454596 CAR T Cell Receptor Immunotherapy Targeting EGFRvIII for Patients with Malignant Gliomas Expressing EGFRvIII	I/II	Biological: CART-EGFRvIII T cells transduced PBL Drug: Aldesleukin Drug: Fludarabine Drug: Cyclophosphamide	Median overall survival was 6.9 months. Two patients survived over one year, and a third patient was alive at 59 months. (doi: 10.1097/CJI.0000000000000260)	National Cancer Institute
	NCT04197934 WSD0922-FU for the Treatment of Glioblastoma, Anaplastic Astrocytoma, or Non-small Cell Lung Cancer with Central Nervous System Metastases	I	Drug: EGFR/EGFRvIII Inhibitor WSD0922-FU Procedure: Therapeutic Conventional Surgery	Recruiting	Mayo Clinic (National Cancer Institute; Wayshine Biopharm, Inc.)
HER2	NCT01109095 CMV-specific Cytotoxic T Lymphocytes Expressing CAR Targeting HER2 in Patients with GBM (HERT-GBM)	I	Biological: HER.CAR CMV-specific CTLs	clinical benefit for 8 of 17 patients (doi: 10.1001/jamaoncol.2017.0184)	Baylor College of Medicine (The Methodist Hospital Research Institute; Center for Cell and Gene Therapy)
	NCT03389230 Memory-Enriched T Cells in Treating Patients with Recurrent or Refractory Grade III-IV Glioma	I	HER2(EQ)BBζ/CD19t+ T cells	Recruiting	City of Hope Medical Center (National Cancer Institute)

#### 2.1.1 IL-13Rα2

IL-13Rα2 as a target for CAR-T in the treatment of GBM. It’s a membrane receptor with a high affinity for the anti-inflammatory cytokine interleukin 13 and it has been discovered to be overexpressed in a variety of solid tumors, most notably GBM, and has been related to poor prognosis (25). IL-13Rα2 is overexpressed in 76% of GBM, but not in the normal brain tissue, which makes it a highly selective target for immunotherapy (26).

In preclinical studies, Christine E. Brown et al. assessed the potential of immunotherapy targeting IL13Rα2 to eliminate GSCs and heavily differentiated populations. This research looked at GSCs as a possible treatment resistance barrier in tumor cells (27). Despite preclinical studies showing that CAR-T cells can produce effective anti-glioma *in situ* mouse models, this approach has not yet been validated in patients. In 2015, they published the first promising human clinical study of intracranially administered IL13Rα2-specific CAR**-**T cells for GBM, which set the stage for the future application of improved peripatetic CAR-T cells therapy (28). This was followed by a case study they published the following year, with the administration of CAR-modified T cells targeting the tumor-associated antigen IL13Rα2 to a patient with recurrent multifocal GBM (29). Regression of all intracranial and spinal tumors was observed subsequent to CAR-T cell therapy, and there was a corresponding increase in cytokine and immune cell levels in the cerebrospinal fluid. Such clinical responses persisted for 7.5 months after the initiation of CAR T-cell therapy (ClinicalTrials.gov, NCT02208362) (29). However, these studies also highlight a few obstacles to achieving more sustained clinical outcomes. First, tumor heterogeneity may promote relapse through the supply of a subsequently scalable pool of target-deficient tumor cells. To address this issue, there is a need to find CAR-T cell approaches that target multiple antigens. Secondly, an absence of persistence of therapeutic CAR-T cells may be another major factor. To address these limitations, Christine E. Brown et al. (30) describe the optimization of IL13Rα-specific CAR-T cells that contain a 4-1BB (CD137) co-stimulatory structural domain (IL13BBζ) to enhance the anti-tumor potency of the IgG4 Fc spacer (L235E, N297Q) and mutation reduction with Fc γ receptor binding. Enhanced anti-tumor activity and T cell persistence in patients with IL13BB-CAR-T cells as compared to first-generation IL13-CAR CD8+ indicates the biological activity of T cells. Given the widespread use of corticosteroids in the clinical care of GBM, they evaluated their effects and found that modest dosages of dexamethasone did not impair the anti-tumor efficacy of CAR-T cells *in vivo*. Local intracranial delivery of CAR-T cells has also been reported to have greater anti-tumor activity than intravenous administration. In another investigation, the antigen-binding domain of newly created IL13Rα2-specific CARs was mutated forms of IL13. Although these CARs target IL13Rα2, they also recognize IL13Rα1, which is broadly expressed. Giedre Krenciute et al. (31) created a set of IL13Rα2-specific CARs with IL13Rα2-specific scFv 47 as antigen-binding domains, short or long spacer regions, transmembrane domains, and intracellular domain molecules derived from co-stimulation and CD3.ζ. In co-culture and cytotoxicity studies, IL13Rα2-CAR T cells detect IL13Rα2-positive target cells but do not cross-react with IL13Rα1. Only IL13Rα2-CAR T cells with a short spacer region, on the other hand, generated IL2 in an antigen-dependent way. T cells expressing IL13Rα2-CAR with a short spacer region and the internal domains CD28.ζ, 41BB.ζ, and CD28.OX40.ζ demonstrated significant anti-glioma activity *in vivo*. Overall, CAR-T cell therapy has the potential to become an effective approach for the clinical management of brain tumors.

#### 2.1.2 EGFRvIII

The epidermal growth factor receptor (EGFR), as the first tyrosine kinase receptor to be cloned, is still at the leading edge of targeted cancer therapy. Being the most common variant of EGFR, EGFRvIII is usually expressed in GBM (32) and is also detected in many epithelial cancers, but not in normal tissues. It is caused by an in-frame deletion in exons 2 to 7 of the EGFR gene and the creation of a new glycine residue at the junction of exon 1 and exon 8. This mutant receptor has constitutive activity in tumors and can lead directly to the cancer phenotype because of its oncogenic nature.

Overexpression of EGFRvIII is considered as a poor prognostic marker, independent of other factors such as age and extent of resection, and may be partly due to its oncogenic nature conferring stability and a persistent tumorigenic signal. Peptide vaccine strategies (rindopepimut) targeting EGFRvIII mutant oncoproteins is a therapeutic approach (33), and secondary immunotherapy using redirected T cells does not require the presentation of antigens and stimulation of primary immune responses and may have more favorable kinetics as compared to vaccines. A neoepitope of EGFRvIII is induced by an in-frame loss of portion of the extracellular structural domain. Based on the success of mouse scFv-based CARs in a GBM xenograft model, Laura A. Johnson et al. (34) chose a vector backbone encoding second-generation CARs. In xenograft subcutaneous and *in situ* models of human EGFRvIII+GBM, EGFRvIII-targeted CAR T cells were also able to suppress tumor development. They also planned a phase I clinical research utilizing humanized scFv-transduced CAR T cells targeting EGFRvIII in patients with residual or recurrent GBM based on these findings (ClinicalTrials.gov, NCT02209376) (35). A first-of-its-kind human study was conducted by Donald M. O’Rourke et al. (35) in which a single dose of autologous T cells was redirected to EGFRvIII mutations by CAR for intravenous infusion. The result showed that single dose of peripherally infused EGFRvIII-directed CAR-T cells mediates antigen loss and induces adaptive resistance in patients with relapsing GBM. However, the major challenges to clinical success of this treatment are the heterogeneity of EGFRvIII expression and the suppressive tumor milieu, which is increasingly immunosuppressive after CAR**-**T cells. The former requires new antigens to be targeted, while the latter may be circumvented by current medications that target immunosuppressive molecules. Animal studies have indicated that an additional 4-1BB co-stimulatory signaling promotes tumor persistence and localization (31), hence the third-generation construct was chosen for clinical trials. Stephanie L. Goff et al. (36) used a third-generation chimeric antigen receptor construct produced from a human antibody in a dose-escalation phase I study in patients with recurrent GBM expressing EGFRvIII (ClinicalTrials.gov, NCT01454596). Anti-EGFRvIII-CAR+ T cells were treated with infusion products in 18 patients. All patients experienced the expected transient hematological toxicity from preparations of chemotherapy, and the median overall survival of patients was 6.9 months, with two patients surviving for more than a year and a third patient surviving for 59 months. The persistence of CAR+ cells correlated with cell dose, but there was no objective response. However, the Administration of anti-EGFRvIII CAR-transduced T cells in this phase I pilot trial did not show a clinically meaningful effect in patients with polymorphic GBM.

#### 2.1.3 EphA2

The EphA2 receptor is a member of the Eph family of receptor tyrosine kinases. Although EphA2 overexpression is a crucial antigen in the maintenance of the malignant GBM phenotype, EphA2 is not expressed in normal brain tissue (37). Targeting EphA2 could prevent tumor immune escape, and Jill Wykosky et al. proposed that EphA2 could be a novel molecular marker and target for GBM (38). CAR-T treatment for GBM must guarantee that such antigens are abundantly expressed on most tumor cells but are generally lacking in normal tissues. Therefore, EphA2 has already proven to be a successful target antigen for CAR-T immunotherapy for GBM. A development of an EphA2-specific CAR was reported by Kevin KH Chow et al. (39) to redirect T cells to EphA2-positive GBM *in vitro* with the aim of identifying and killing EphA2-positive glioma cells and glioma-initiating cells, as well as inducing tumor induction in an *in situ* xenografted GBM with Severe Combined Immunodeficiency (SCID) mouse model of regression. However, such manipulations are still carried out through highly artificial means and may not better predict future clinical effectiveness. H.T. Lin et al. (40) conducted the first human study of EphA2-redirected CAR T cells in EphA2-positive recurrent GBM patients. A single intravenous infusion of EphA2-redirected CAR T cells was combined with a lymphocyte clearance regimen of fludarabine and cyclophosphamide. Two patients had grade 2 cytokine release syndrome with pulmonary edema, which was entirely cured with dexamethasone medication therapy, with most cytokines reverting to normal as the edema dropped. The pulmonary edema observed in these patients may be due to an “on-target, off-tumor” effect. However, the possibility of “off-target, off-tumor” lung organ cytotoxicity cannot be completely ruled out in the study. There were no additional organ toxicities, including neurotoxicity. They detected CAR T cell growth in peripheral blood and cerebral fluid for more than four weeks. The tumour shrank metastatically in one patient. One patient had stable disease, while the other two had progressing disease, with an overall survival of 86 to 181 days. At the dose level tested, the intravenous infusion of EphA2 redirected CAR T cells was initially tolerated with transient clinical efficacy. Future research will be needed to modify the dose and frequency of CAR T cell infusions. Zhongzhen Yi et al. (41) showed the anti-tumour effectiveness of third-generation CAR-T cells targeting distinct EphA2 epitopes against GBM. While there have been substantial advances in the clinical effectiveness of EphA2 redirected CAR-T cells for GBM, the anti-tumour effects of CAR-T cells generated in different labs or by different methods remain uneven. Several parameters, including target antigen affinity, off-target toxicity and terminal differentiation, could influence the anti-tumour effects of CAR-T cells, therefore future research on CAR-T cells against GBM are still in its early stages.

#### 2.1.4 HER2

HER2, an epidermal growth factor receptor protein encoded by the ERBB2 gene, has been found to be over-expressed as a tumour-associated antigen in 80% of GBM cells, signaling a poor prognosis, but not in normal neurons or glial cells (42). HER2 is now being aggressively targeted as a cell surface protein in GBM-directed CAR-T cell treatments in preclinical models. Nabil Ahmed et al. (43) identified HER2-specific T cells to target primary GBM stem cells and induce autologous experimental tumour regression. In a phase I clinical trial, Nabil Ahmed et al. (44) revealed that the infusion of autologous HER2-specific embedded CAR-modified virus-specific T cells (VST) is safe and is potentially linked to clinical benefit in patients with progressive GBM (ClinicalTrials.gov, NCT01109095). These cell lines are enriched with cytomegalovirus, Epstein-Barr virus and adenovirus. In this study, they determined the safety of autologous HER2-CAR VST in 17 patients with progressive GBM, with no serious adverse events. 8 patients showed clinical benefit, with a median overall survival of 11.1 months after T-cell infusion and 24.5 months after diagnosis, and 3 patients were alive at last follow-up with no disease progression. However, the efficacy of HER2-CAR VST as a single agent or in combination with other immunomodulatory approaches for the treatment of GBM needs to be further evaluated in phase 2b studies.

#### 2.1.5 Multi-antigen targeted CAR-Ts

Regardless of the major breakthroughs in clinical efficacy, every CAR-T therapy has certain drawbacks when it comes to treating GBM (30, 35, 41, 44). This is due in large part to the fact that safe, specific and homogeneously expressed targets are more difficult to identify, which suggests that there are few antigens that are truly tumour-specific and consequently the cross-reactivity of engineered T cells with normal tissues for targeting/non-tumour can lead to lethal toxicity (45–48). Rather, these targets are often heterogeneously expressed even when antigens with high tumour specificity are identified, and selective CAR targeting can allow antigen-negative tumour cells to escape (35). GBM is a prime example of this dual challenge, and several of these issues that have impeded the efficacy of CAR**-**T cells need to be addressed during the treatment of GBM. Therefore, in recent years, more and more CAR**-**T therapies targeting multiple antigens have been proposed, thus avoiding the problems of tumour specificity and heterogeneity associated with single CAR-T therapies. As revealed by Masasuke Ohno et al. (49), expression of MicroRNA (miR) -17-92 augments the anti-tumour activity of T-cells transduced with the anti-EGFRvIII chimeric antigen receptor in mice bearing human GBM xenografts. Meenakshi Hegde et al. (50) used two glioma antigens, HER2/IL-13Rα2 bivalent T-cell products, both of which counteracted antigen escape and enhanced T-cell effector function. However, site-specific antigen pairs are variably different between patients and therefore require the generation of permutations of bivalent T-cell products, which would make the successful clinical translation of this approach challenging. Kevin Bielamowicz et al. (51) created for the first time a trivalent T-cell product, i.e. a single CAR**-**T cells product using 3 targetable glioma antigens (HER2, IL13Rα2 and EphA2) for broader application. Trivalent CAR**-**T cells have the potential to overcome antigen heterogeneity in GBM and improve treatment results. Furthermore, Joseph H. Choe et al. (52) hypothesized that T cells recognizing various antigen combinations give a potential solution to the issue of maximizing tumour identification specificity and killing integrity at the same time. SynNotch receptors that identify particular priming antigens in GBM (53), such as the highly tumour-specific GBM neoantigen EGFRvIII or the CNS tissue-specific antigen myelin oligodendrocyte glycoprotein (MOG), can be employed to homogeneously trigger CAR production in tumors. EGFRvIII expression in tumors is extremely selective, and while CAR**-**T cells successfully destroy EGFRvIII+ tumour cells, EGFRvIII- tumour cells can escape and thrive (34, 35, 49). EGFRvIII has specificity but is heterogeneous, as opposed to EphA2 and IL13Ra2, both of which are more homogenous but only partially specific for tumors. Due to their unique flaws, it is possible that these antigens be coupled to form a multi-antigen circuit with both high specificity and the ability to cause death more comprehensively. In addition, because they lacked co-localized priming antigens, EGFRvIII synNotch-EphA2/IL13R2 CAR**-**T cells were able to efficiently and completely eliminate GBM tumors without destroying surrounding normal tissue or EphA2 or IL13R2-positive cells elsewhere in the body. They also discovered that T cells carrying α-MOG synNotch receptor may be effectively and selectively activated in the CNS body by endogenously produced MOG (54, 55). If these cells are driven to produce α-EphA2/IL13R2 CAR, they will only kill CAR-expressing cells in the CNS, not those transplanted outside the CNS. Ultimately, through the use of circuits incorporating recognition of multiple imperfect but complementary antigens, the specificity, integrity and persistence of the T cells targeted to GBM were improved, and therefore, they managed to provide a general recognition strategy applicable to other solid tumors.

### 2.2 NEs therapy

Neutrophils (NEs), the most prevalent leukocyte population in the blood, may be rapidly recruited to sites of inflammation, and are thought to be a powerful platform for tumour-targeted drug delivery, similar to mesenchymal stromal cells (MSCs) (56). More importantly, NEs can also penetrate the BBB/BTB and specifically brain tumour sites. Inflammation can activate NEs and is often accompanied by a local inflammatory response in the brain after surgical resection of GBM, with massive release of inflammatory factors. Therefore, the inflammatory TME may be a promising therapeutic strategy for GBM. Nanoparticle-based drug delivery systems (DDSs) are seen as a promising prospective technique for brain-targeted medication delivery (57). Although it has demonstrated the capacity to improve tumour targeting, these DDSs cannot accomplish the full therapeutic potential of postoperative glioma therapy because the predominant distribution of particles is in the perivascular region of recurring tumors and because intratumoural drug concentrations are low (58, 59). Xue Jingwei et al. (60) created a cell-based anti-cancer DDS that uses the physiological features of natural NEs to improve the efficacy of postoperative glioma therapy. Unlike conventional nanoparticles, their accumulation at the tumour site is based on passive targeting, i.e. increased permeability and retention effects, or active targeting *via* ligand-receptor interactions. NE-mediated DDS have the ability to recognize post-operative inflammatory signals such as IL-8 and CXCL1/KC and deliver chemotherapeutic agents to infiltrating glioma cells in a spontaneous and on-demand manner. They used cationic liposomes carrying paclitaxel (PTX) as a delivery vehicle based on NEs to effectively deliver PTX to tumour cells and induce cytotoxicity and inhibit post-operative recurrence of GBM (60). Furthermore, highly concentrated inflammatory signals in the brain after surgery triggered NEs to release liposomal PTX, thus allowing the delivery of PTX to the remaining invasive tumour cells. This suggests that this NE-mediated drug delivery is effective in slowing down recurrent tumour growth. Meiying Wu et al. (56) internalized doxorubicin-loaded magnetic mesoporous silica nanoparticles (D-MMSNs) loaded with the antitumor drug Adriamycin (DOX) into inflammation-activatable neutrophils. It provides magnetic resonance imaging (MRI) to track drug-loaded cells and actively target inflamed brain tumors after surgical removal of the primary tumour, releasing D-MMSNs to be taken up by infiltrating GBM cells, so as to maximize the bioavailability of the drug for accurate diagnosis and high anti-glioma efficacy. However, it has been shown that NEs can be polarized into different functional phenotypes in the TME, while it can also be polarized into N1-type anti-tumour or N2-type pro-tumour phenotypes, i.e. the controversy of having both pro- and anti-tumour effects (61–63). The antitumor activity of N1 TAN includes the expression of more immune activating cytokines and chemokines, reduced arginase levels, and a greater ability to kill tumour cells *in vitro*. As blockade of TGF-β facilitates the accumulation of N1 TAN with antitumor activity, TGF-β is the major proximal cytokine within tumors that defines the TAN phenotype and biases differentiation towards the N2 pro-tumorigenic phenotype (62). To address this controversy, Jun Wang et al. (59) mounted DOX into neutrophil exosomes (NEs-Exos), which are extracellular vesicles with characteristics of NEs. It can produce a chemotactic response to inflammatory stimuli and target infiltrating tumour cells in inflamed brain tumors without the risk of tumour promotion. This is an addition to the current research on NEs for GBM.

### 2.3 Immunotherapy of MSCs

**Figure 3:**
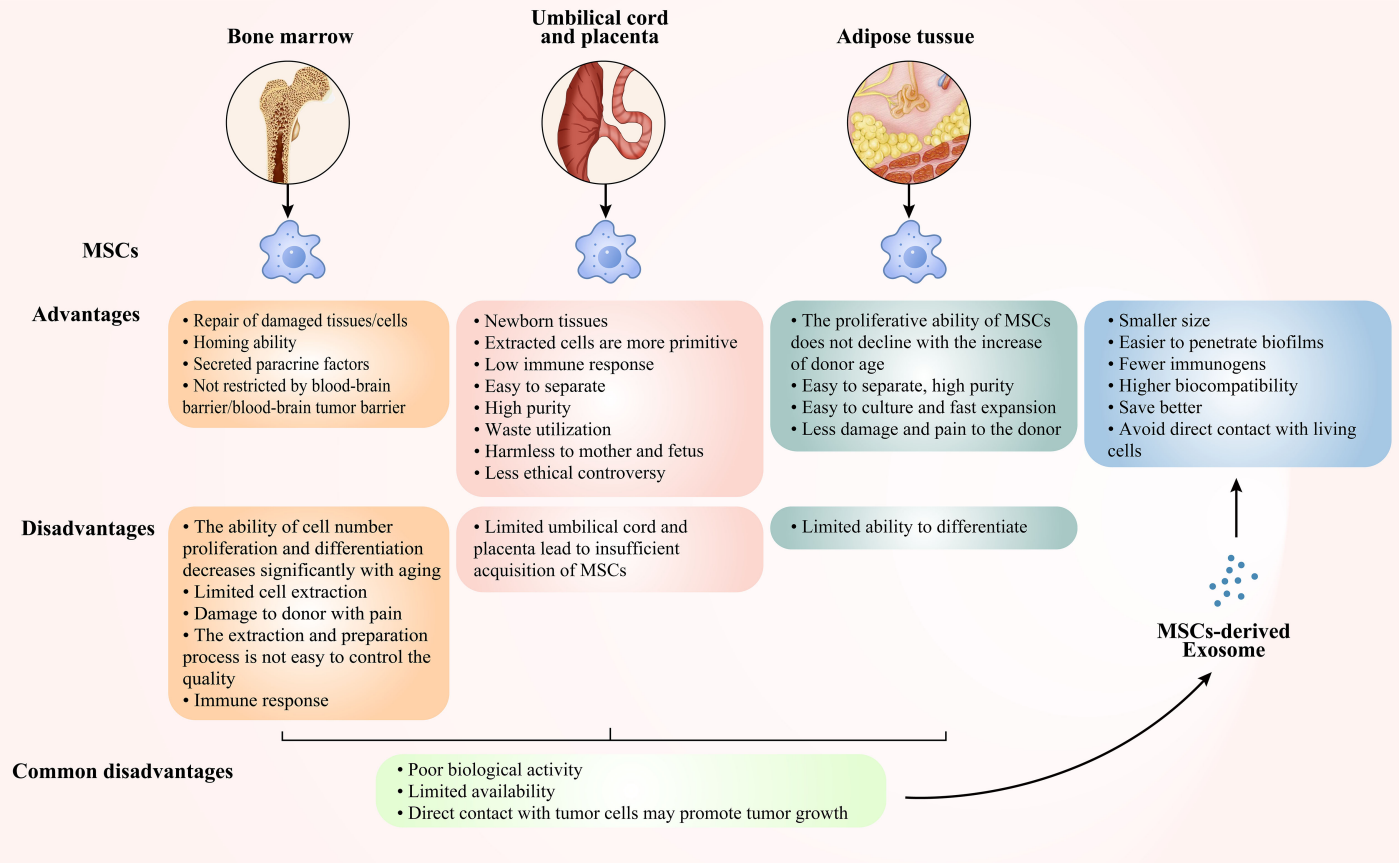
Advantages and disadvantages of bone marrow-derived, cord- and placenta-derived, adipose-derived MSCs and MSCs-derived Exo.

MSCs are pluripotent stem cells, which are normally derived from bone marrow, umbilical cords/placenta, and adipose tissue. MSCs have tissue healing capability and low immunogenicity, and they are not restricted by the BBB/BTB, so they can be intrinsically subsumed into the brain tumour site (64, 65), overcoming the difficulties of conventional therapy being isolated by the BBB/BTB. That is, MSCs exhibit tropism to the cytokines, chemokines and growth factor-mediated TME. Studies have shown that MSCs and their derived soluble factors exhibit inhibitory effects on the growth of GBM cells, revealing a well-established role for MSCs in the treatment of CNS malignancies (66). Nevertheless, there are certain benefits and drawbacks of MSCs generated from different tissues (**Figure 3**), and we will explore the mechanism by which MSCs derived from diverse tissues prevent the development of GBM in recent years.

#### 2.3.1 Bone marrow-derived MSCs

MSCs were first identified in bone marrow and their tumour tropism has been used for the delivery of anti-cancer therapeutic genes, but MSCs exact mechanisms in the TME remain unknown. Vy A W Ho et al. (67) investigated the biological effects of MSCs from bone marrow on glioma cells. MSCs limit tumour angiogenesis by releasing anti-angiogenic factors, according to their findings. Further studies using antibody array technology showed reduced expression of platelet-derived growth factor (PDGF)-BB, IL-1β, phosphorylated Akt and histone B proteins in MSCs/glioma co-cultures. In conclusion, their findings imply that the antitumor actions of MSCs may be mediated by down regulation of the PDGF/PDGFR axis, which is important in glioma angiogenesis. Based on the fact that bone marrow-derived MSCs have been shown to localize to gliomas after intravascular delivery and that these cells are located in inflammatory regions of tissue injury in the TME, Jonathan G. Thomas et al. (68) used ionizing radiation (IR) to increase the tropism of bone marrow MSCs towards GBM. IR is a therapeutic modality that can effectively trigger local damage or inflammation in the TME. According to their results, IR to GSC-derived gliomas increases MSCs tropism, which can be boosted by the chemokine CCL2. Nevertheless, IR can increase vascular permeability by disrupting the BBB (69–72), reduce tight junction proteins (73) or induce endothelial cell damage (74), leaving the mechanism of action of IR in an *in vivo* situation where tumour cells are integrated with supportive cells more unknown. Improving the efficiency of MSCs in the treatment of GBM requires the use of appropriate tools and technical abilities. Extensive research has revealed the promise of suicide genes in the treatment of glioma tumors. Enhancing their effectiveness relies on the ability to apply the right tools and techniques. Saeed Oraee-Yazdani et al. (75) investigated the safety and feasibility of treating patients with primary and secondary polymorphic GBM with lentiviral transduced autologous bone marrow MSC containing herpes simplex virus thymidine kinase (HSV-TK) in combination with intravenous ganciclovir. From the five patients they recruited, it was possible to find that all patients had a 1 year progression-free-survival (PFS) and overall survival (OS) of 60% and 100%, respectively, and two patients had an OS of more than 3 years and a PFS of more than 2 years. This finding suggests that intracerebral injection of bone marrow MSCs expressing the HSV-TK gene, in conjunction with intravenous ganciclovir is safe and practical for treating GBM patients. When recurrent cell infusions are necessary in the same patient, autologous cell sources are frequently used. Some studies have discovered that bone marrow MSCs from healthy donors can be viral carriers (76). However, it is unknown if bone marrow MSCs can be created from chemotherapy-treated glioma patients, or whether such bone marrow MSCs can successfully transmit oncolytic virus. Yuzaburo Shimizu et al. (77) conducted a prospective clinical experiment in which they discovered that bone marrow MSCs could be collected from GBM patients who had previously had chemotherapy and that bone marrow MSCs were efficient carriers of oncolytic virus. Additionally, Nazneen Aslam et al. (78) suggested a possible solution for GSCs and discovered that when actively developing GSCs were treated with paracrine factors from MSCs, the prospective growth capacity and pluripotent of GSCs were disrupted. This effect was mediated by up regulation of the DKK1 gene, which in addition was mediated by up regulation of the Wnt pathway mediated by inhibition of growth factor activity and down regulation of the KITLG gene activated by growth factor and cytokine activity, thus exhibiting antitumor properties. The main active component of paracrine secretion is extracellular vesicles (EVs), which will be discussed in the Cell-free Immunotherapies for the GBM section. Even so, the proliferation and differentiation capacity of bone marrow-derived MSCs in terms of cell numbers decreases considerably with age, thus leading to limitations in cell extraction. It is also harmful to the donor, the extraction and preparation process is difficult to achieve quality control, and transplantation into humans may trigger an immune response (79, 80). These issues have hampered the therapeutic use of bone marrow MSCs.

#### 2.3.2 Umbilical cord or placental-derived MSCs

The umbilical cord and placenta are novel tissues and the cells removed are primitive. As the cells are young, the functional activity of cell surface antigens is low, making it difficult to stimulate an immune response. It has also been shown to be a waste product that does not cause any harm or damage to the mother or newborn when collected and has a greater capacity for proliferation and differentiation (80–83), so it may be an ideal alternative to bone marrow-derived MSCs. Based on the fact that MSCs exhibit tropism towards cytokines, chemokines and growth factor-mediated TME. Adriana Bajetto et al. (84) examined the effect of umbilical cord MSCs on the growth of GSCs. Umbilical cord MSCs released large amounts of soluble cytokines regarding inflammation, angiogenesis, cell migration and proliferation, such as IL-8, GRO, ENA-78 and IL-6. They regulate GBM cells, either through direct cell-to-cell interactions or indirectly. These cytokine ligands share a receptor, CXC chemokine receptor 2 (CXCR2), so they also assessed the effect of CXCR2 on the proliferation of GSCs induced by umbilical cord MSCs. The results showed that direct (intercellular contact) or indirect (via release of soluble factors) interactions between GSCs and umbilical cord MSCs in co-culture had different effects on anti-GSCs, with the former causing mainly an inhibitory response and the latter a stimulatory response involving paracrine activation of CXCR2. miRs are promising therapeutic targets for GBM, but the difficulties in delivering them to tumour target cells has limited their usage. MSCs can migrate to cancer sites, including GBM, and exert antitumor effects. S Sharif et al. (85) found that delivery of exogenous miR-124 to GBM cells *via* umbilical cord-derived MSCs reduced cell proliferation and migration and conferred sensitivity to the chemotherapeutic agent TMZ. To explore the potential clinical application of gadolinium neutron capture therapy (Gd-NCT) in GBM treatment affected by the rapid clearance and non-specific bio-distribution of gadolinium-based drugs, Yen-Ho Lai et al. (86) developed a stem cell-nanoparticle system (SNS) that actively targets GBM by using gadobisamine-concealed magnetic nanoparticles (Gd-FPFNP) on umbilical cord-derived MSCs was performed to actively target GBM for advanced Gd-NCT. The findings of their study indicate that SNS can potentially overcome the current limitations of Gd-NCT, including off-target effects and rapid metabolism, and that it combines the advantages of cellular therapy and nanotechnology for an alternative strategy to treat brain disorders. However, umbilical cord-derived and placental-derived MSCs are limited in availability, which limits their clinical application (80, 87).

#### 2.3.3 Adipose tissue-derived MSCs

The proliferative potential of adipose tissue-derived MSCs does not reduce with increasing patient age and is less harmful and uncomfortable to the donor (81). Most significantly, because it is easy to extract and has a high potential for self-renewal, adipose tissue-derived MSCs are thought to be a viable alternative source of therapeutic stem cells (87). Mona N. Oliveira et al. (88) emphasized the processes through which adipose-derived MSCs interact with GBM cells, with substantial implications for MSCs in the treatment of GBM. MSC-based gene delivery of tumour necrosis factor-related apoptosis-inducing ligand (TRAIL) is recognized as a potent therapy for GBM (89, 90). The systemic treatment of TRAIL-secreting stem cells is problematic in that some of these delivery vehicles do not always reach tumour microsatellite nests. Furthermore, as many stem cells are home to normal brain parenchyma and perivascular gaps, TRAIL-laden stem cells are unable to reach tumour microsatellite nests, causing them to remain in normal brain tissue and cause adverse effects such as neuronal cell death. To regulate the expression of suicide inducers and reduce off-target damage, Man Li et al. (91, 92) exploited endogenous tumour signaling pathways to modulate the release of the suicide inducer TRAIL. Findings from their study suggested a significant improvement in the efficacy of adipose MSC-mediated gene delivery for the treatment of GBM. Bahattin Tanrikulu et al. (93), Valentina Coccè et al. (94) also found that the combination of TRAIL-expressing adipose MSCs and multiple drugs (e.g. X-linked apoptosis protein (XIAP) inhibition, XIAP silencing, and octane diamide isohydroxamic acid) or paclitaxel induced GBM cell apoptosis and reduced their proliferation. To improve the effectiveness of adipose-derived MSCs to reach the actual tumour target, Francesco Agostini et al. (95) utilized growth factor-rich supernatant as an additive to adipose MSCs. The results showed that the growth factor-rich supernatant enhanced the specific homing and secretory properties of adipose MSCs towards GBM. However, the ability of adipose-derived MSCs to differentiate into cells is relatively limited.

Despite the numerous benefits of MSCs in the battle against GBM, there are some drawbacks to their use, such as poor biological activity and restricted availability. Additionally, when MSCs come into direct touch with GBM cells, they not only do not operate as tumour suppressors, but instead accelerate tumour development (96). These restrictions add to the inherent dangers of MSCs as live cells.

The ability to homing is critical for MSCs to be employed safely and successfully in therapeutic applications. However, many systemically administered MSCs are lost in patients’ substantial organs such as the lungs, liver, and spleen, significantly reducing MSCs’ therapeutic usefulness. If they are given a “GPS” to guide them to their final destination, off-target effects can be minimized. To address this problem, research have looked at using pro-inflammatory cytokines (IL-7, IL-12, and TNF-α) and chemokines (CXCR1, CXCR4) to better recruit MSCs to GBM sites (97, 98). In addition to this, the targeting of MSCs to the GBM can be improved by targeting target genes that are specifically highly expressed in the GBM or highly heterogeneous. For example, Irina V Balyasnikova et al. (99) found that MSCs genetically engineered to express EGFRvIII on the cell surface had increased affinity for GBM target sites. TRAIL is one of the few anti-cancer proteins that selectively causes apoptosis in tumour cells by activating death receptors, while having no effect on healthy cells. Xiang-Jun Tang et al. (100) found that MSCs carrying TRAIL exerted sustained anti-GBM activity. In another preclinical study, MSCs armed with both EGFR-targeting nanoantibodies (ENb) and TRAIL were evaluated to significantly improve the survival of animals in a GBM *in situ* resection model (101). OV selectively replicates and kills cancer cells and spreads within the tumour without harming normal tissue. It also promotes the release of tumour-associated antigens, activates antigen-presenting cells, promotes the activation and aggregation of CD4+ and CD8+ T cells, and directly kills tumors (26). The use of MSCs as a delivery vehicle helps protect the virus from the immune system and improves therapeutic efficiency by enhancing tumour shrinkage (102). Delta-24-RGD is a tumourolytic virus who’s binding to MSCs has been shown to selectively target intra-arterially delivered hMSCs-Delta24 to GBM and to deliver and release Delta-24-RGD into tumors, thereby improving survival and tumour eradication in a subpopulation of mice (76). MSCs loaded with oHSV induced significant anti-GBM mechanisms in preclinical models or GBM resection (103). MSCs enhance the tumourolytic effect of Newcastle disease virus on GBM and GSC cells through the secretion of TRAIL (104).

### 2.4 NK cells therapy

Natural killer (NK) cells play an essential role in the body’s anti-infection, anti-tumour, and immunomodulatory processes as recognition and effector cells in the innate immune system. NK cells do not inhibit the killing of their own normal cells, but selectively recognize and kill cells that are low in expression or lack their own major histocompatibility complex (MHC)-I molecules (105). MHC-I molecules are also known as human leukocyte antigen class I (HLA-I) molecules. The binding of killer cell immunoglobulin-like receptors (KIRs) on the surface of NK cells to HLA-I on the surface of target cells induces inhibition of NK cell killing activity (106, 107). When there is a lack of expression of HLA-I on the surface of target cells, the killing activity of NK cells against them can be triggered. The imbalance between KIRs and HLA-I has been shown to trigger NK cells to successfully destroy glioma cells (108). Furthermore, NK cells activity is governed by a variety of signaling factors, and Hila Shaim et al. (109) discovered that GSCs are susceptible to *in vitro* destruction by healthy allogeneic NK cells. Their findings demonstrated that GBM tumour-infiltrating NK cells acquired an altered phenotype associated with impaired lytic function when compared to matching peripheral blood NK cells from GBM patients or healthy donors. This immune evasion approach was attributed to αv integrin-mediated TGF-β activation, which directed interactions between GSCs and NK cells. In contrast, blocking the αv integrin/TGF- β axis can increase NK cell antitumor function. Gregory J Baker et al. (110) found that NK elimination of intracranial neuroGBM was possible in the presence of decreased tumour-derived galactose lectin 1 (gal-1).

The function of monoclonal antibodies like bortezomib and bevacizumab in the antitumor process is getting attention. Andrea Gras Navarro et al., Thi-Anh-Thuy Tran et al. (111, 112) discovered that pretreatment of GBM with the monoclonal antibody bevacizumab increased NK cell cytotoxicity and extended animal life. Relay transfer of CAR-modified NK cells has shown significant anti-glioma activity both *in vitro* and *in vivo*. In contrast to CAR-T cells, which require autologous cells for each patient, NK cells are safe under allogeneic circumstances, which broadens the pool of cell donors capable of producing therapeutically meaningful amounts of CAR-NK cells for therapy (113). In terms of safety, CAR-NK cells outperform CAR-T cells because they operate autonomously on antigen-antibody reactions and do not produce cytotoxic effects, such as cytokine release syndrome, in different studies (114). The inherent characteristics of NK cells make them an appealing option to CAR-engineered effectors in cancer treatment, clearing the way for several clinical studies to further develop the strategy and better its ability to fight glioma cells (114, 115). CAR-NK cells, in brief, identify CAR-targeted antigens and induce NK cell activation, proliferation, and secretion of different inflammatory cytokines and chemokines. When NK cells recognize cancer cells, they establish lytic synapses with them to guide the delivery of lytic granules to susceptible cancer cells while maintaining their normal activating and inhibitory receptors (115). Thus, CAR-NK cells can kill cancer cells that do not exhibit CAR-targeting antigens (CAR non-dependent) as well as cancer cells that do express CAR-targeting antigens (CAR dependent) (116).

### 2.5 DC cells therapy

Dendritic cells (DC) are now recognized as the most potent and only specialist antigen-presenting cells in the body capable of activating naïve T cells. It is called after the numerous dendritic protrusions that emerge from the cell surface during maturation. Immature and mature dendritic cells perform distinct tasks, with immature dendritic cells being good at antigen differentiation and phagocytosis and mature dendritic cells being good at antigen presentation. DC have MHC class I and II molecules on their surface, which when combined with antigenic peptides trigger CD4+ helper T cells (Th) and CD8+ cytotoxic T lymphocytes (CTL) to elicit specific anti-tumour cellular and humoral immunity, inhibiting tumour cell growth (117). DC also upregulate the expression of cell surface co-stimulatory factors such as MHC-II, CD80, CD86 and CD40, and secrete cytokines such as interleukins and chemokines to stimulate T cell activation, proliferation and aggregation, thereby inducing the activation of an adaptive immune response.

DC vaccines, which are currently being studied extensively in anti-tumour immunotherapy, are based on the powerful antigen-presenting ability of dendritic cells. The key to developing and manufacturing DC vaccines is to allow dendritic cells to carry a marker for the target tumour, which can come from a variety of sources, such as tumour antigen gene modifications (118), synthetic antigenic peptides, antigen-encoded mRNA or DNA (119), which DC then deliver to T cells. Immune cells such as killer T cells can then accurately and effectively recognize and assault the target tumour cells, significantly decreasing collateral harm to normal cells. According to research, recombinant adenovirus-mediated gene transfer is the most efficient way of changing DC cells (120). GBM secrete a range of immunosuppressive and immune escape factors, such as substantial loss of Fas, to avoid immune killing initiated by the Fas/FasL system (121, 122). Mature dendritic cells improve antigen presentation, activating the Fas/FasL-mediated apoptotic pathway and increasing Fas mRNA, causing a caspase enzyme chain reaction that results in planned cell death. Dendritic cells have no direct killing impact, but they improve immunosurveillance and tumour suppression by improving antigen expression (123). Furthermore, studies have shown that NK and other cells have a glioma-killing impact (124), implying that there is still much space for study into the inhibitory effect of DC-associated killer cells on gliomas. Xin Ma et al. and Haidar A Shamranet al. (125, 126) discovered that glioma cells secreted immunosuppressive factors VEGA and IL-10, which reduced immune cell function, meanwhile Yawen Ma et al. (127) discovered that miR-153 can down-regulate VEGA expression in malignant glioma vessels, inhibiting tumour growth. The first two clinical studies involving DC cell vaccines for the treatment of high-grade gliomas were reported (128). Surasak Phuphanich et al. (129) assessed the findings of a phase I clinical study of the autologous DC cell vaccine ICT107. The vaccine was pulsed with class I peptides from six tumour-associated antigens (TAA) of AIM-2, MAGE1, TRP-2, gp100, HER2/neu and IL-13Rα2, which are expressed on gliomas and over expressed in their cancer stem cell population. The feasibility, safety, and biological efficacy of a TAA-pulsed dendritic cell vaccination in patients with GBM were proven in this phase I study of ICT-107. Based on PFS and OS measures in newly diagnosed GBM patients, AIM2 and MAGE1 antigen expression in pre-vaccination tumors was related to longer survival, whereas HER2 and gp100 expression exhibited a trend toward prolonged PFS and OS. They are conducting a randomized, placebo-controlled phase II study based on these positive results. Patrick Y Wen et al. (130) published the findings of a phase II clinical study in which ICT-107 improved the immunosuppressive microenvironment in newly diagnosed GBM and helped patients overcome tumour heterogeneity, but there was no advantage in terms of total patient mortality (ClinicalTrials.gov, NCT01280552). HLA-A2 primary tumour antigen expression was more frequent than in HLA-A1 patients. HLA-A2 patients had a higher immune response (by Elispot) and patients in the pre-specified subgroups of methylated and unmethylated MGMT achieved meaningful therapeutic benefit with ICT-107. This was the first vaccination study to demonstrate a clinical benefit in GBM, and it paved the way for a phase III trial in patients with HLA-A2+ newly diagnosed GBM. Linda M Liau et al. (131) published interim results from a phase III clinical trial of the autologous tumour cell lysate-loaded dendritic cell vaccine DCVax-L in combination with TMZ dendritic cell vaccine, and the phase III trial results showed promising application (ClinicalTrials.gov, NCT00045968) (**Table 2**). They later reported that including DCVax-L in standard of care (SOC) resulted in a clinically relevant and statistically significant extension of life for patients with GBM when compared to concurrent (132). The use of autologous DC vaccines pulsed with allogeneic GBM or GBM stem cell line lysates for the therapy of freshly identified and recurring GBM is also safe and well accepted, according to Jethro L Hu et al. and Ian F Parney et al. (133, 134). The above clinical findings contribute to the evidence that immunotherapy may play a part in the treatment of GBM.

**Table 2:** Dendritic cells based clinical studies in GBM patients that have been completed or are ongoing.

Molecular target	Clinical trial NCT number and title	Study phase	Interventions	Study Results	Sponsor/Collaborators
DC	NCT01280552 A Study of ICT-107 Immunotherapy in Glioblastoma Multiforme (GBM)	II	Biological: ICT-107 Biological: Placebo DC	The ICT-107 vaccination was well tolerated, with a 2.2-month improvement in progression-free survival. Overall survival, the primary outcome, was not improved. (doi: 10.1158/1078-0432.CCR-19-0261)	Precision Life Sciences Group
	NCT02546102 Phase 3 Randomized, Double-blind, Controlled Study of ICT-107 in Glioblastoma	III	Biological: ICT-107 Biological: Placebo	Suspended	Precision Life Sciences Group; Medelis Inc.
	NCT03014804 Autologous Dendritic Cells Pulsed With Tumor Lysate Antigen Vaccine and Nivolumab in Treating Patients With Recurrent Glioblastoma	II	Biological: autologous dendritic cells pulsed with tumor lysate antigen Vaccine Other: Laboratory Biomarker Analysis Biological: Nivolumab Other: Quality-of-Life Assessment Other: Questionnaire Administration	Withdrawn	Jonsson Comprehensive Cancer Center; Northwest Biotherapeutics Bristol-Myers Squibb Brain Tumor Funders Collaborative
	NCT00045968 Study of a Drug [DCVax®-L] to Treat Newly Diagnosed GBM Brain Cancer	III	Drug: Dendritic cell immunotherapy	As of this analysis, 223 patients are ≥ 30 months past their surgery date; 67 of these (30.0%) have lived ≥ 30 months and have a Kaplan-Meier (KM)-derived mOS of 46.5 months. Only 2.1% of ITT patients (n = 7) had a grade 3 or 4 adverse event that was deemed at least possibly related to the vaccine. doi: 10.1186/ 2236 s12967-018-1507-6	Northwest Biotherapeutics

### 2.6 Microglia and Tumour-associated macrophages

Microglia and tumour-associated macrophages (TAMs) are the main components of GBM myeloid cells, which are maintained by self-renewal under physiological conditions and are associated with functions such as CNS inflammation and development (135). Under pathological conditions, especially in GBM, GBM cells release multiple chemokines, such as MCP-1 and CCL2, which allow microglia to activate and accumulate in large numbers around the tumour. At this point, BBB/BTB function is hampered, and monocytes in the blood also penetrate the brain parenchymal *via* the impaired BBB/BTB, and both cells are converted into critical drivers of tumour development by acting as TAMs together to infiltrate at GBM locations (136).

TAMs are the most common immune cells in the TME, and their phenotype is diverse and flexible (137). The bulk of macrophages in tumors are Tumour-promoting TAMs (pTAMs), which interact tightly with tumour cells and thus support tumour growth. pTAMs have the characteristics of M2 macrophages, which are M2 TAMs that support tissue healing and remodeling, Th2 immune response, and tumour progression, and generate Arg-1, IL-10, and TGF-β. pTAMs are the primary factors to the development of an immunosuppressive microenvironment in tumors (138). TAMs are a subset of macrophages in tumors that phagocytose or destroy tumour cells, thereby inhibiting tumour development (139). TAMs have M1 macrophage characteristics, and M1 TAMs exhibit high amounts of pro-inflammatory factors (e.g. TNF-α) and anti-tumour factors IL-12, IL-13, IL-1, and TNF-β, which can boost Th1 responses and tumour-killing capability. Because many malignancies, including GBM and brain metastases, contain significant quantities of tumour growth-promoting pTAMs, recoding pTAMs into sTAMs is a novel approach to successful cancer control and therapy. Wenchao Zhou et al. demonstrated that GSCs can greatly decrease the capacity of TAMs to attract TAMs by silencing periostin (POSTN) secretion, thereby inhibiting tumour development (140). GSC-secreted granulocyte-macrophage colony-stimulating factors (GM-CSFs) produced CD11+ macrophages, a subset of CD11c (high) cells with tumour-promoting activity, according to Yasuhiro Kokubu et al. (141).

## 3 Cell-free Immunotherapies for GBM

### 3.1 MSCs-derived exosomes as carriers

Despite the many advantages of MSCs in the fight against GBM, there are some limitations to the use of MSCs, such as low biological activity and limited accessibility (79, 80, 87). Furthermore, in direct contact with GBM cells, MSCs enhance the development of tumors rather than inhibit them (96). This argument highlights the inherent danger of MSCs as live cells. EVs are cell-secreted nanoparticles with a bilateral lipid membrane structure that are actively released by the cell. Based on their biogenesis, size and biophysical properties, the types of EVs can be classified as microvesicles, apoptotic vesicles and exosomes. Microvesicles, approximately 100-1000 nm in diameter, generated by cells directly outwards budding or extruding from cells, containing cell membranes and some cytoplasmic components (142, 143). Apoptotic vesicles, which range in size from 50 nm to 5000 nm, are vesicles shed or burst during apoptosis or death and released outside the cell (144, 145). Distinct from microvesicle formation, exosomes (Exo), which are approximately 30-100 nm in diameter, begin at the cell membrane and bud inwards to produce intracellular vesicles, then undergo early intracellular vesicles, multivesicular complexes, directed assembly and migration, and finally fuse with the cell membrane and depart the cell by exocytosis (146, 147). Exo have the lowest average particle size, the greatest mean content, and the most diversified roles among the three kinds of extracellular vesicles.

MSCs can be an abundant source of Exo, and all Exo express the same group of proteins, such as tetraspanins (e.g. CD63, CD9, CD81), adhesion proteins (e.g. L1CAM, LAMP2), Alix and TSG101 (148–150). Exo vesicle proteins are closely related to proteins in the source cells, such as heat shock proteins (HSP70, HSP90) and cellular skeletal proteins (actin, tubulin, cofilin) (151). Apart from this, Exo carries the same bioactive substances as the source cells, such as nucleic acids, proteins and lipid substances, and can produce a variety of biological effects (152–154).

MSCs exosomes (MSCs-Exo), as paracrine mediators of the therapeutic effect of MSCs, have comparable biological activity to MSCs. However, compared with MSCs, MSCs-Exo are smaller, penetrate biological membranes more easily, are less immunogenic, more biocompatible, and better preserved (155, 156). Previous studies have shown that exosomes are important mediators of intercellular communication. It can be used as carriers of drugs/signaling molecules to efficiently transport cargo to target cells (157, 158). Therefore, MSCs-Exo can be used to safely and effectively deliver drugs to GBM sites in the brain. MSCs-Exo preferentially homed to damaged tissues and sites of inflammation, including brain malignant gliomas (159, 160). This suggests that these exosomes, like the MSCs from which they are formed, could be used as potential new therapeutics. Furthermore, they provide considerable advantages over uncontrolled cell development and potentially tumour formation in live cells due to their ability to decrease severe side effects and infusion toxicity (96, 161–163). Many studies have demonstrated that microRNAs (miRs) such as miR-93 (164), miR-519a (165), miR-758-5p (166), miR-330-5p (167), miR-139-5p (168), miR-590-3p (169), miR-34a (170, 171) may inhibit GBM production. Unfortunately, the lack of an ideal delivery system has limited the clinical application of miRs in the fight against GBM. Several studies on GBM (including GSCs) have established the transport of GBM-inhibitory miRs to tumour sites through MSCs-Exo to limit tumour development (172–178), suggesting that MSCs-Exo have significant potential for application in the treatment of GBM. Pharmacological delivery to treat GBM has been unsatisfactory, mainly attributed to drug resistance and low targeting efficiency. A combination of selective targeting of GBM cells and synergistic induction of apoptosis using a cocktail of therapeutic agents may help to improve drug delivery. Rana Rahmani et al. (179) found that treating GBM cells with modified MSCs-Exo with two apoptosis-inducing gene therapy agents, cytosine deaminase (CDA) and miR-34a, and targeting the EGFRvIII antigen, enhanced the rate of apoptosis.

### 3.2 Oncolytic virus

With the advancement of scientific research, not only cellular therapy and Exo are used for tumour immunotherapy but also oncologic viruses (OV) have become effective new therapeutic tools in this field (180–182). OV is a class of naturally occurring or genetically engineered viruses that may infect or kill tumour cells while without harming normal cells. OV has been divided into two types, mildly virulent strain of wild-type OV/natural OV, represented by reovirus, retroviruses and poliovirus (183, 184), and a strain that has been genetically modified to proliferate only within tumour cells, such as adenovirus and herpes simplex virus (185, 186). OV exerts anti-tumour activity *via* several mechanisms. At first, viruses proliferate in tumour cells and directly lyse tumour cells (187). Then, lyses of tumour cells lead to the release of newly generated viral particles that stimulate systemic anti-tumour immune responses through a variety of pathways, such as promoting tumour antigen presentation, improving the immunosuppressive TME, disrupting the tumour vascular system and stimulating adaptive immune responses (188–191). Due to space constraints, this section concentrates on OV therapy of GBM using Reovirus, adenovirus, and herpes simplex virus (**Table 3**).

**Table 3:** Oncolytic virus based clinical studies in GBM patients that have been completed or are ongoing.

Molecular target	Clinical trial NCT number and title	Study phase	Interventions	Study Results	Sponsor/Collaborators
oHSV-1	NCT03152318 Treatment of Recurrent Malignant Glioma With rQNestin34.5v.2 (rQNestin)	I	Drug: rQNestin Drug: Cyclophosphamide	humans with recurrent GBM treated with rQNestin34.5v.2 has not shown evidence of viral-mediated toxicity or encephalitis (doi: 10.1016/j.omtm.2020.03.028; doi: 10.1158/1078-0432.CCR-21-2347)	Dana-Farber Cancer Institute (National Institutes of Health; Candel Therapeutics, Inc.)
	NCT02457845 HSV G207 Alone or With a Single Radiation Dose in Children with Progressive or Recurrent Supratentorial Brain Tumors	I	Biological: G207 Single dose of HSV-1 (G207) infused through catheters into region(s) of tumor defined by MRI	a total of 4 of 11 patients were still alive 18 months after G207 treatment (doi: 10.1056/NEJMoa2024947)	University of Alabama at Birmingham (Food and Drug Administration; National Center for Advancing Translational Sciences of the National Institutes of Health; et)
	NCT04482933 HSV G207 With a Single Radiation Dose in Children with Recurrent High-Grade Glioma	II	Biological: Single dose of HSV-1 (G207) infused through catheters into region(s) of tumor defined by MRI	Not yet recruiting	Pediatric Brain Tumor Consortium (Treovir, LLC)
	NCT03911388 HSV G207 in Children with Recurrent or Refractory Cerebellar Brain Tumors	I	Single dose of G207 infused through catheters into region(s) of tumor. If G207 is safe in the first cohort of patients, subsequent patients will receive a single dose of G207 infused through catheters into region(s) of tumor followed by a 5 Gy dose of radiation to the tumor within 24 hours of virus inoculation.	Recruiting	University of Alabama at Birmingham
OAds	NCT03896568 MSC-DNX-2401 in Treating Patients with Recurrent High-Grade Glioma	I	Biological: Oncolytic Adenovirus Ad5-DNX-2401 Procedure: Therapeutic Conventional Surgery	The use of perfusion guidance to enhance the precision of endovascular super-selective intra-arterial infusions of mesenchymal stem cells loaded with Delta-24 in the treatment of GBM (doi: 10.1136/neurintsurg-2021-018190)	M.D. Anderson Cancer Center (DNAtrix, Inc.)
	NCT03072134 Neural Stem Cell Based Virotherapy of Newly Diagnosed Malignant Glioma	I	Biological: Neural stem cells loaded with an oncolytic adenovirus	The post-treatment PES and OS of 12 newly diagnosed malignant glioma patients were 9.05 months and 18.4 months, respectively (doi: 10.1016/S1470-2045(21)00245-X)	Northwestern University (National Cancer Institute)
	NCT02197169 DNX-2401 With Interferon Gamma (IFN-γ) for Recurrent Glioblastoma or Gliosarcoma Brain Tumors (TARGET-I)	I	Drug: Single intratumoral injection of DNX-2401 Drug: Interferon-gamma	The addition of IFN did not improve survival, but clinical activity following a single injection of DNX-2401 is encouraging (doi: 10.1200/JCO.2017.35.15_suppl.2002)	DNAtrix, Inc.
Reovirus	NCT02444546 Wild-Type Reovirus in Combination with Sargramostim in Treating Younger Patients with High-Grade Relapsed or Refractory Brain Tumors	I	Biological: Wild-type Reovirus Sargramostim	All patients progressed on therapy after a median of 32.5 days and died a median of 108 days after recruitment (doi: 10.1093/noajnl/vdac085)	Mayo Clinic (National Cancer Institute)

#### 3.2.1 Herpes simplex virus-1

Oncolytic herpes simplex virus (oHSV-1) is a neurophilic double-stranded DNA virus. Typically, wild HSV-1 is neurotoxic, so the virus must be genetically modified or greatly attenuated to ensure safety. After genetic modification, OV can still maintain its ability to reproduce while replicating specifically in tumour cells, and therefore OV is widely exploited. Based on previous findings, oHSV was the first state-of-the-art genetically engineered OV to be licensed by the United States FDA for cancer treatment (192) and was approved for the treatment of advanced melanoma (193, 194).

However, as GBM is a primary brain tumour of the human central nervous system, more attention deserves to be paid to the safety of oHSV-1 in the fight against GBM. In past studies, oHSV-1 is encoded by the γ34.5 gene, an ICP34.5 protein, which is neurotoxic (195). To limit neurotoxicity, the double copy γ34.5 gene was knocked out in all oHSV-1 tested in glioma clinical trials, the first generation of oHSV-1. But replication of γ34.5-deficient oHSV-1 is often restricted and severely attenuated, particularly in GSC (196, 197). It is crucial to assure the 34.5 deletion mutant’s safety while also ensuring its effective replication in GBM. Hiroshi Nakashima et al. (198) produced the gene HSV-1 OV (NG34), an attenuated HSV-1 with the deletion of the gene encoding the viral ICP6 gene (UL39) and the gene for γ34.5. The UL39 gene encodes the large subunit ICP6 of ribonucleotide reductase, which is essential for postmitotic cell replication. GADD34 gene in humans is expressed by NG34 under the transcriptional regulation of the cellular Nestin gene promoter/enhancer element, which is specifically expressed in GBM. In a GBM mouse model, they discovered that the new oHSV encoding GADD34 was efficacious and generally non-toxic. Another research found that activating MEK in tumour cells boosted replication of γ34.5-deficient HSV-1 (199), but activating MEK in tumour-associated macrophages (TAM) stimulated pro-inflammatory signaling while inhibiting viral replication and propagation (200). Ji Young Yoo et al. (201) investigated the effects of blocking MEK signaling and oHSV-1 binding on brain tumors. It was reported that oHSV treatment facilitated the entry of the MEK kinase inhibitor trametinib into brain tumors and sensitized it *in vivo*.

G207 is a second generation oHSV-1 that inactivates the ICP6 gene by deleting the double copy γ34.5 gene while inserting the lacZ gene at the UL39 locus. During a Phase I clinical trial of genetically engineered oHSV-1 G207 by GK Friedman et al. (202), oHSV-1 G207 was found to establish an effective anti-tumour immune response in pediatric high-grade gliomas (ClinicalTrials.gov, NCT02457845). Subsequently, to extend and confirm the results of this phase I trial, an upcoming multi-institutional phase II clinical trial of G207 in pediatric high-grade glioma (ClinicalTrials.gov, NCT04482933) is still under investigation.

G47Δ is a third generation oHSV-1 with a triple mutation in α47 deleted from the G207 genome. Compared to G207, G47Δ is further attenuated in normal cells, but has enhanced efficacy in anti-tumour, as well as a greater safety profile. oHSV-1 G47Δ showed efficacy and safety in GBM was confirmed by the American Association for Cancer Research in 2016 (203). In subsequent years, Tomoki Todo et al. (204) published their findings of a phase I/II single-arm study in 2022 evaluating the safety of G47Δ for the treatment of recurrent/progressive GBM (ClinicalTrials, UMIN000002661). These findings support and formed the basis of a phase II clinical study in patients with GBM. This was followed by a separate report evaluating G47Δ in residual or recurrent GBM, that is, a phase II trial that revealed a survival benefit and a good safety profile, leading to the approval of G47Δ as the first Japanese OV product for the treatment of GBM.

#### 3.2.2 Oncolytic adenovirus

Adenovirus is a non-enveloped virus with an icosahedral capsid containing approximately 38 kb of genomic double-stranded DNA. There are more than 50 serotypes of adenovirus in humans, of which types 2 and 5 have been most frequently studied for the manufacture of lysing viruses (205, 206). In addition to its high genetic stability, high titer production and its ease of purification, the 38kb capacity of the adenovirus capsid allows for the introduction of large transgenes and as a result adenoviruses have been genetically engineered into various types of oncolytic adenovirus (OAds) or conditionally replicating adenovirus (CRAd). Just like other types of OV, OAds are able to replicate relatively specifically in tumour cells and lyse them, releasing progeny viruses that then infect surrounding tumour cells and destroy the tumour through a cascade amplification effect, so that a better outcome can be achieved.

Several treatments for OAds are currently in clinical trials, including Frederick F Lang et al. (207) in a phase I clinical trial of OAds DNX-2401 (Delta-24-RGD), where 20% of patients (5 of 25), survived > 3 years after treatment, three patients had ≥ 95% (12%) reduction in enhancing tumors, and and > 3 years progression-free survival from the start of treatment. This demonstrates that DNX-2401 is safe and has anti-tumour activity in patients with GBM. DNX-2401 is a potential second generation OAds with significant viral replication capacity and the ability to directly destroy tumour cells. DNX-2401 is a potential second-generation OAds with significant viral replication capacity and direct tumour cell destruction. Its mechanism of anti-tumour action is that a 24pb base deletion in the E1A gene that prevents it from binding the retinoblastoma tumour suppressor protein (Rb) protein and thus from replicating in normal tissues, whereas in Rb-deficient tumour cells, where E2F is in a free state, the virus can still replication (208). As the primary mode of entry of adenovirus type 5 into host cells is through binding to coxsackievirus and adenovirus receptors on the cell surface, which are expressed at low levels on the surface of GBM cells (209), DNX-2401 was designed with an RGD peptide insert, Delta-24-RGD, which has an RGD-4C peptide motif inserted into adenovirus fibers, and allows viral entry *via* the integrins avβ3 and avβ5 into tumour cells, which further enhances tumour targeting (210). To increase the anti-glioma immune effect of Delta-24-RGD, in a preclinical study, Yisel Rivera-Molina et al. (211) decided to arm Delta-24-RGD with co-stimulatory ligand glucocorticoid receptor-enhanced T-cell activity (GITRL) with the aim of activating the T-cell population recruited to the tumour after viral infection. From their data, GITRL-armed Delta-24-RGD exhibited enhanced anti-glioma effects, resulting in an increased frequency and activation of T cells. In addition, specific anti-tumour immunity and enhanced central T cell memory encouraged preclinical testing of next generation lysing adenoviruses equipped with immune checkpoint modulators. Given the safety of DNX-2401 in past studies (207), through a phase I clinical trial on convection-enhanced delivery of Delta24-RGD in the tumour and peripheral brain of patients with recurrent GBM, Erik HP van Putten et al. (212) showed that 19 out of 20 enrolled patients received the oncolytic adenovirus Delta24-RGD, which was considered safe and feasible. Four patients demonstrated tumour response on MRI, and one of them regressed completely and is still alive 8 years later. This trial was the first to assess local and regional responses to the injection of OV into the tumour and surrounding brain by serial sampling of interstitial fluid and cerebrospinal fluid (CSF). Analysis of cytokines and chemokines in CSF suggests that IFNγ and TNFα levels may represent potential biomarkers of response in future OV assays. Biomarker testing may ultimately help to identify patients who respond and improve response rates to OV therapy. Their findings show promising clinical responses and indications for anti-tumour immune responses, providing a basis for future testing of (combined) Delta24-RGD treatments in GBM.

ICOVIR17 is an OAds expressing soluble PH20 hyaluronate (HA) that degrades HA and spreads efficiently in the tumour. It has the same mechanism of action as Delta-24-RGD, ICOVIR17 deletion of 24 base pairs in the Rb-binding domain of E1A for tumour-selective replication and RGD modification in the fibril to amplify tropism, except for two additional modifications: insertion of an E2F binding site in the E1A promoter and insertion of the SPAM1 gene encoding PH20 HA after the fibril, which is controlled by the major late promoter control (213). Normally, adenovirus replication is divided into two phases, early (E) and late (L). Early stage expresses adenovirus replication-related genes E1-E4, and late stage expresses adenovirus assembly-related genes L1-L5. However, OAds, as well as most novel targeted therapies, face significant transport barriers in the tumour mesenchyme, in part because they are relatively large (90 nm) and much larger than chemotherapeutic agents. Solid tumors exhibit unique features that impede the transport of large molecules. Among these, the large amount of extracellular matrix present in the tumour mesenchyme and high mesenchymal fluid pressure are the main sources of physical resistance to drug transport. HA is an essential component of the ECM with high expression in most tumour xenografts. Jordi Martinez-Quintanilla et al. (214) revealed for the first time that intratumoural injection ICOVIR17 into nodal GBM mediated HA degradation and enhanced viral dissemination, resulting in significant anti-tumour effects and mouse survival. As much as this work reveals that HA functions in GBM as a physical barrier to effective virus dissemination and tumour killing, it remains unknown whether HA affects the immune response induced by OAds treatment of brain tumors as the mice used in the study were immunodeficient. Therefore, Juri Kiyokawa et al. (215) exploited that degradation of HA would enhance OAds immunotherapy of GBM by overcoming the immunosuppressive function of GBM extracellular matrix. In their study, murine GBM 005 was chosen as a suitable *in vivo* model given that this GBM model encapsulates key features of human disease, including GSC properties and immunosuppressive TME. Their study has shown for the first time that immunomodulatory ICOVIR17 has the dual role of mediating HA degradation in GBM extracellular matrix and subsequently altering the TME immune landscape, and provides a mechanistic combination of immunotherapy and PD-L1/PD-1 blockers to remodel innate and adaptive immune cells.

CRAd-S-pk7, a type of oncolytic adenovirus, is a promoter doped with survival proteins to drive expression of the replication-essential E1A gene and modifies Ad5 fibronectin by doping with a polylysine sequence (pk7) (216, 217). These modifications enhanced viral replication and targeting of glioma cells, resulting in enhanced antitumor activity and higher survival rates *in vivo*. Jawad Fares et al. (218) conducted the first human phase I dose-escalation trial investigated NSC-CRAd-S-pk7, a CRAd delivered by neural stem cells, for use in patients with newly diagnosed GBM. Their findings support the continuation of the study of NSC-CRAd-S-pk7 in a phase II/III clinical trial. In addition, multi-dose neural stem cell viral therapy (NSC-CRAd-S-pk7) for recurrent high-grade glioma is being investigated (ClinicalTrials.gov, NCT05139056).

#### 3.2.3 Reovirus

Reovirus (RV) belongs to the wild-type OV family of eutheroviruses and is characterized as a staged double-stranded RNA virus. The three prototypical serotypes of RV, first identified in the 1960s, are well characterized as serotype 1 Lang (T1L), serotype 2 Jones (T2J) and serotype 3 Dearing (T3D) (219). RV is widely present in the respiratory and digestive tracts of humans and livestock without causing disease and is only associated with mild flu-like symptoms. RV has been shown to have a tumourolytic effect on a variety of tumour cells and has been used in several clinical trials (220–223). Among these, Peter Forsyth et al. (224) demonstrated for the first time in a single institution phase I clinical trial that intratumoural injection of wild-type eutherian virus in GBM patients was well tolerated. Kimberly P Kicielinski et al. (225) in a preliminary study of direct intratumoural inoculation of the CNS, once again they observed that RV well tolerated: patients had a median progression-free survival of 4.3 weeks and a median survival of 21 weeks. In the GBM study and other previous studies, the tolerability of RV at the dose administered prompted them to design a clinical trial of incremental viral doses intended to achieve higher doses and better distribution of study drug. With the safety and tolerability demonstrated in several phase I clinical trial studies, RV embarked on a phase II study to assess efficacy, particularly in areas where GBM treatment was not effective.

### 3.3 Immune checkpoint inhibition

GBM generates an immunosuppressive environment through multiple mechanisms, including the programmed cell death protein 1 (PD-1), cytotoxic T lymphocyte antigen 4 (CTLA-4), lymphocyte activating gene 3 (LAG-3) pathway (226). Although some tumors benefit from immune checkpoint inhibition (ICI), GBM does not (24).

The immunosuppressive properties of the GBM microenvironment lead to immune evasion by tumour cells, making inhibition of immune checkpoints such as PD-1 alone ineffective (226, 227). PD-1 inhibition is thought to disrupt the binding of PD-1 to its inhibitory ligands, thereby stimulating cytotoxic T cell-mediated tumour elimination. The Ivy Foundation Early Clinical Trials Consortium conducted a multi-institutional, randomized clinical study to evaluate immunological response and survival in 35 patients with recurrent, surgically resectable GBM after neoadjuvant anti-PD-1 immunotherapy with pembrolizumab (228). Pembrolizumab is an anti-PD-1 monoclonal antibody that has been demonstrated to be effective as a monotherapy in a variety of cancer types, but largely in adjuvant treatment (229). OS was significantly longer in patients randomized to neoadjuvant pembrolizumab and continuing adjuvant therapy after surgery. Neoadjuvant PD-1 inhibition was linked with increased T-cell and interferon-γ (IFN-γ) related gene expression, but decreased intratumor cell cycle-related gene expression, which was not observed in patients receiving adjuvant treatment alone. Local induction of programmed death ligand 1 (PD-L1) in the tumour microenvironment, increased T cell clonal expansion, decreased PD-1 expression in peripheral blood T cells, and decreased monocyte numbers were more frequently observed in the neoadjuvant group in patients treated. These data imply that neoadjuvant PD-1 blocker administration boosts local and systemic antitumor immune responses and may be a more effective therapy for this consistently deadly brain tumour. In the single-arm phase II clinical trial (ClinicalTrials.gov, NCT02550249) by Kurt A Schalper et al. (230), preoperative doses of nivolumab followed by postoperative nivolumab were tested in 30 patients (27 recurrent cases for salvage surgery and 3 newly diagnosed patients for initial surgery) until disease progression or unacceptable toxicity. Neoadjuvant nivolumab resulted in enhanced expression of chemokine transcripts, increased immune cell infiltration and enhanced TCR clonal diversity in tumour-infiltrating T lymphocytes, supporting a local immunomodulatory effect of the treatment, although no clear clinical benefit was demonstrated after salvage surgery.

#### 3.3.1 Simultaneous inhibition of multiple immune checkpoints

Simultaneous inhibition of multiple immune detection sites for anti-GBM treatment may improve treatment outcomes. Antonio Omuro et al. (226) evaluated the anti-PD-1 checkpoint inhibitor nivolumab intravenously in patients with recurrent GBM in a phase I trial, both as monotherapy or in combination with CTLA-4 blocking mAb ipilimumab at different dose levels. Nivolumab as a single agent is well-tolerated, but the combination of nivolumab and ipilimumab is associated with up to 30% of treatment-related adverse events that lead to treatment discontinuation. The tolerability of the combined treatment was determined by the dose of ipilimumab. In two patients who were initially identified as having suspected progression based on neuroradiological assessment and subsequently underwent neurosurgical resection, interestingly, substantial immune cell aggregates were identified by histopathological examination, but no live tumors. John Lynes et al. (231) used cytokine micro dialysis to detect real-time immune assays in GBM patients undergoing PD-1 and LAG-3 checkpoint inhibition, suggesting that the anti-LAG-3 and anti-PD-1 combination may have a similar immune response side effect profile to other checkpoint inhibitor combinations. E Antonio Chiocca et al. (232) reported an increase in tumour-infiltrating lymphocytes producing IFN-γ and PD-1 in a phase I trial. These inflammatory infiltrates support the immune anti-tumour effects of human interleukin 12. E Antonio Chiocca et al. (233) found a reduction in PD-1 and/or PD-L1 positive cells in four pre- and post-treatment biopsy matched subjects in their trial. This validates the hypothesis that nivolumab reduces GBM cell immune checkpoint signaling induced after treatment with controlled IL-12 gene. In addition, Moreover, recruitment has been completed for a phase II study of controlled IL-12 in combination with neoadjuvant anti-PD-1 (ClinicalTrials.gov, NCT04006119). However, as O^6^-methylguanine-DNA methyltransferase (MGMT) renders tumors resistant to TMZ, MGMT promoter status predicts both prognosis and therapeutic response to TMZ chemotherapy. Antonio Omuro et al. (234) conducted CheckMate-498 phase III clinical study comparing nivolumab or TMZ for OS, each in combination with radiotherapy (RT), in patients with newly diagnosed MGMT unmethylated GBM, failed to meet its intended target improvement OS endpoint (ClinicalTrials.gov, NCT02617589). In GBM, the entry of monoclonal antibodies (mAb) is blocked due to CNS is an immune-privileged site. In tumour types, combined treatment with two mAb leads to higher tumour response rates and improved survival compared to monotherapy for the cost of serious immune-related adverse events (235–239). Johnny Duerinck et al. (240) in a phase I clinical trial Intracerebral administration of CTLA-4 and PD-1 immune checkpoint blocking monoclonal antibodies in patients with recurrent GBM. The phase III randomised CheckMate 548 study by Michael Lim et al. (241) identified that nivolumab added to RT+TMZ was not associated with improved survival in newly diagnosed GBM patients with a methylated or indeterminate MGMT promoter. Although these studies failed to demonstrate the clinical benefit of ICI, they could be considered in new combination therapy strategies.

#### 3.3.2 Immune checkpoint inhibition binding to oncolytic virus

Possible complementary effects on tumour killing through combination with OV. Dipongkor Saha and colleagues in 2018 GBM may be treated with oHSV immunoviral therapy in combination with two checkpoint inhibitors (anti-PD-1 and anti-CTLA-4), a triple combination that could assist in curing less immunogenic malignancies such as GBM (31). Also based on the fact that OV and PD-1 inhibitors have become standard immunotherapies against certain cancers, Carmela Passaro et al. (36) conducted preclinical trials in 2019 on GBM, i.e. they investigated *in vitro* and *in vivo* the efficacy of a novel lysine virus (NG34scFvPD-1) of HSV-1 against PD-1, which also expresses a single-chain fragment mutable antibody (scFvPD-1). Irene Appolloni et al. (65) also investigated the specificity, safety and efficacy of EGFRvIII-targeted oHSV-1 for the treatment of human GBM. These studies provide a basis for further exploration of this novel OV in combination with ICI for cancer therapy.

## 4 Future perspectives and conclusions

There is a deepening perception of what cell-based and cell-free immunotherapy can bring to GBM, which offers new hope for GBM patients, but many details and questions remain to be explored and elucidated. According to recent statistics, adjuvant immunotherapy can prolong survival and significantly improve outcomes for patients with recurrent GBM compared to those treated with only surgery, radiotherapy or chemotherapy. To summarize the lessons learned, the extremely specific and heterogeneous nature of GBM, as well as the complicated immune resistance mechanisms and immunosuppressive TME have resulted in relatively limited response effectiveness and durability of response to treatment drugs. The BBB/BTB is another barrier to medication delivery. These are still tough breakthroughs in GBM therapy. We not only obtained some novel findings on the theoretical study of the local immune characteristics of glioma, but we also provided an experimental basis for the comprehensive diagnosis and treatment of regulating and intervening in the immune microenvironment of glioma. More significantly, “supporting the righteousness” and improving the immune microenvironment, in conjunction with “elimination of evil” by anti-tumour cells, will ideally decrease disease mortality rates, extend patient life, and genuinely help patients. In fact, the treatment of GBM is actually a complex “project” and satisfactory results can hardly be achieved with only one treatment. Immunotherapy research for GBM should be coupled with other treatment modalities in future anti-GBM research, resulting in truly tailored and complete care strategies for patients. It is recommended to develop combined treatment techniques based on immunotherapy, molecular targeted therapy, and radiation to maximize therapeutic efficiency and reduce acquired immunotherapy resistance. The industry therefore needs to innovate, integrate and translate to drive immune combinations forward in a sustained manner. Secondly, assessing therapeutic response to immunotherapy is difficult, and in the future, a standardized imaging and molecular biology evaluation system will be required to reflect and forecast patient outcomes. Cell-based and cell-free immunotherapy are predicted to become an essential component of future glioma treatment when these challenges are solved.

## Author contributions

MW conceived this article. MW and XW drafted the manuscript. MW and XW drew the illustrations and tables. MW, XW, XJ, JZho and YZ revised the article. MW, YL and JZha checked and edited the article. YL and YY supervised the article. All authors have read and agreed to the final version of the manuscript.
